# The Basis for Natural Multiresistance to Phage in *Pseudomonas aeruginosa*

**DOI:** 10.3390/antibiotics9060339

**Published:** 2020-06-18

**Authors:** Christine Pourcel, Cédric Midoux, Gilles Vergnaud, Libera Latino

**Affiliations:** Institute for Integrative Biology of the Cell (I2BC), CEA, CNRS, Université Paris-Saclay, 91198 Gif-sur-Yvette, France; cedric.midoux@irstea.fr (C.M.); gilles.vergnaud@universite-paris-saclay.fr (G.V.); libera.latino@gmail.com (L.L.)

**Keywords:** bacteriophage, *Pseudomonas aeruginosa*, coevolution, mechanisms of resistance, genome sequence, prophage induction, mobile genetic elements, CRISPR-Cas systems, lateral transduction, phage therapy

## Abstract

*Pseudomonas aeruginosa* is responsible for long-term infections and is particularly resistant to treatments when hiding inside the extracellular matrix or biofilms. Phage therapy might represent an alternative to antibiotic treatment, but up to 10% of clinical strains appear to resist multiple phages. We investigated the characteristics of *P. aeruginosa* clinical strains naturally resistant to phages and compared them to highly susceptible strains. The phage-resistant strains were defective in lipopolysaccharide (LPS) biosynthesis, were nonmotile and displayed an important degree of autolysis, releasing phages and pyocins. Complete genome sequencing of three resistant strains showed the existence of a large accessory genome made of multiple insertion elements, genomic islands, pyocins and prophages, including two phages performing lateral transduction. Mutations were found in genes responsible for the synthesis of LPS and/or type IV pilus, the major receptors for most phages. CRISPR-Cas systems appeared to be absent or inactive in phage-resistant strains, confirming that they do not play a role in the resistance to lytic phages but control the insertion of exogenous sequences. We show that, despite their apparent weakness, the multiphage-resistant strains described in this study displayed selective advantages through the possession of various functions, including weapons to eliminate other strains of the same or closely related species.

## 1. Introduction

*Pseudomonas aeruginosa* is a Gram-negative bacterial species ubiquitous in the environment and associated with different types of infections in the hospital. Resistance to antibiotics is at the origin of the emergence of clones that are widely distributed worldwide and are responsible for nosocomial infections [1,2]. Interestingly, long-term infections in cystic fibrosis or burn patients are not systematically related to multidrug resistance but, rather, to the capacity of the strains to evolve and hide in biofilms [3,4]. The wide genetic diversity of the species is favored by the plasticity of the genome mediated by mobile genetic elements (MGE) including integrative conjugative elements (ICE), genomic islands (GI), and insertion sequences (IS) which are segments of DNA acquired by horizontal gene transfer (HGT) [5,6]. Prophages constitute another class of mobile elements. It has been estimated that, in addition to a highly conserved core genome, strains possess specific sequences that can account for 10 to 20% of their genome and therefore are considered as “accessory” [7,8,9,10]. The accessory genome is subject to rapid change in response to selective pressure [11] and plays an important role in antibiotic resistance [12,13]. Regions of Genome Plasticity (RGP) were defined by Mathee et al. as strain-specific regions made of at least four open reading frames (ORFs): firstly RGP01 to RGP62 [11], extended to RGP94 in additional studies (RGP63 to RGP80 [14], RGP81 to RGP89 [15] and RGP90 to RGP94 [16]). New RGPs are continuously discovered in newly sequenced genomes. A large number of RGPs are immediately adjacent to tRNA or tmRNA genes known to act as integration sites for plasmids, phages and genomic islands [5,7,15,17,18]. Prophages and ICE carrying antibiotic resistant genes, metabolic and virulence factors represent a significant part of the accessory genome in *P. aeruginosa* [13,19]. Some phages contribute to the pathogenicity of their host either by carrying virulence factors or by modifying chromosomal gene expression [20,21]. They also provide resistance to other phages through different mechanisms, including the role of moron genes [22,23].

The continuous coevolution of bacteria and phages leads to the existence of strains with very diverse susceptibly reflecting different evolutionary histories [24]. Phages are exerting a strong pressure on their host, which may result in the selection of a variety of mutations, including deletions of large genomic regions allowing the bacteria to resist infection [25,26,27]. Bacteria have developed multiple ways to resist phages [28,29,30]; however, mutations in receptors are the most frequently observed events in laboratory-derived phage-resistant mutants [31]. Whereas temperate phages usually show a restricted host range and affinity for specific receptors, virulent phages of Gram-negative bacteria often require the presence of common structures, principally type IV pili (T4P), lipopolysaccharide (LPS) or O-antigen, for binding to their host. The interactions between the phage tail fiber and the receptor are specific to a given strain. Nonspecific innate immunity is provided by restriction-modification (R-M) systems, toxin-antitoxins and abortive systems. The adaptive immune system constituted by Clustered Regularly Interspaced Short Palindromic (CRISPR) elements and CRISPR-associated (*cas*) genes is unique in that it stores a memory of invasive DNA in the form of short sequences called spacers [32]. In *P. aeruginosa*, about 30% of strains were shown to possess a type I-F CRISPR-Cas system, whereas 6% also possessed a type I-E system [33,34,35]. Some *P. aeruginosa* strains also carry a third CRISPR subtype (type I-C) [35]. Interestingly, the majority of spacers for which the origin is known correspond to temperate phages, plasmids or transferable ICEs [35].

We previously reported that 10% of *P. aeruginosa* isolates from cystic fibrosis patients were resistant to bacteriophages belonging to seven different genera [34]. A similar observation was made using a panel of strains representing the diversity across *P. aeruginosa* and eight lytic phages belonging to four genera [36]. By comparison to the resistance to antibiotics, such strains can be referred to as “multiphage-resistant or MPhR”. Conversely, some strains isolated from similar environments showed a high susceptibility to phages, such as reference strain PAO1 [37,38] or strain PcyII-10 [27]. A multiphage infection of PAO1 in vitro led to the emergence of resistant variants with reduced fitness and deficiency in T4P and LPS expressions [38,39]. It is believed that the fitness cost of such resistant bacteria would be so high in a natural environment that they would not persist [40]. While a loss of receptors present on the appendages necessary for motility such as pili and flagella, outer membrane proteins or LPS will decrease virulence and the capacity to survive [41], mucoid variants or variants secreting higher levels of virulent factors will be more aggressive [38,42,43]. There is still much to learn about the fate of phage-resistant bacteria in patients and in the environment and, particularly, in biofilms [44,45]. This will be of importance when considering phage therapy [46,47].

We describe here the characteristics and genome sequences of three clinical MPhR strains and compare them to PcyII-10, a highly susceptible strain [27]. The phage-resistant strains have accumulated mobile genetic elements predicted to provide selective advantages and further increase their capacity to survive treatments using antibiotics or phages.

## 2. Results

### 2.1. Characteristics of Multiphage-Resistant Strains

More than 200 *P. aeruginosa* strains from our collection originating from cystic fibrosis (CF) and burn patients were tested for their susceptibility to ten phages belonging to different genera and which use as primary receptors eitherT4P, LPS or specifically O-antigen (Table S1). The phage collection included LeviOr01, a Levivirus [48], and PhiKZ, a giant phage [49]. Serial dilutions of the phage were spotted on bacterial lawns. Nineteen strains were not lysed by any phage (9.5%), whereas at the opposite of the spectrum, 15 strains were susceptible to six phages or more (7.5%). To test whether the resistant bacteria were expressing common phage receptors, absorption assays were performed with six virulent phages (Ab01, Ab05, Ab09, Ab17, Ab22 and Ab27, belonging to different genera and which used either LPS or T4P as receptors), and none of them attached to the MPhR strains, suggesting that the receptors were absent or hidden.

**Figure 1:**
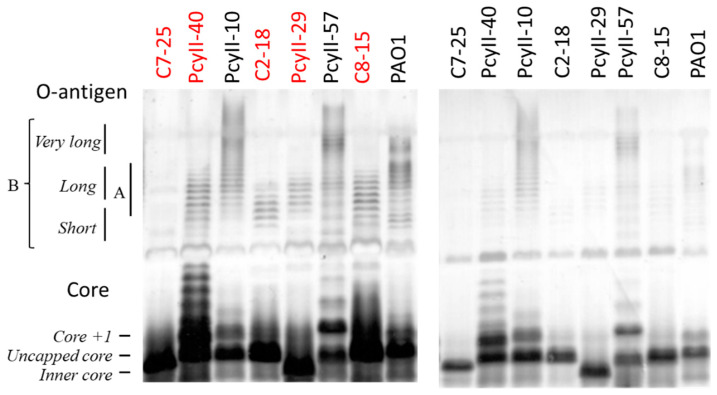
SDS-PAGE of the lipopolysaccharide (LPS) fraction. SDS-PAGE of the LPS fraction. The position of the core, the A-band and the B-band of the O-antigen were identified according to the work of Islam et al. [50]. In order to clearly detect the different molecular species present in the LPS profiles, we had to use different amounts of samples when looking at the long O-chains (left panel) and at the shorter core LPS (right panel). We used 12.5 µg of suspended lyophilized bacteria for the gel on the left panel and 2.5 µg for the gel on the right panel. The resistant strains are shown with red letters.

Next, the LPS of five MPhR strains (PAC2-18, PAC7-25, PAC8-15, PcyII-29 and PcyII-40) and three susceptible strains (PAO1, PcyII-10 and PcyII-57) were extracted and analyzed by sodium dodecyl sulfate (SDS) polyacrylamide gel electrophoresis (PAGE) (Figure 1). In PAO1, short and long O-antigen B chains were observed, whereas PcyII-10 and PcyII-57 apparently lacked short B chains but possessed very long B chains. All the resistant strains lacked long and very long B chains while possibly possessing short B chains, which migrate like A chains. Consequently, they were O-antigen-defective, explaining why these cells were not typable. Concerning the core on which the O-antigen is attached, PcyII-29 possessed only inner core species, whereas PAC2-18, PAC7-25 and PAC8-15 produced mostly uncapped cores. The core +1 band in PcyII-40 was very abundant.

**Figure 2:**
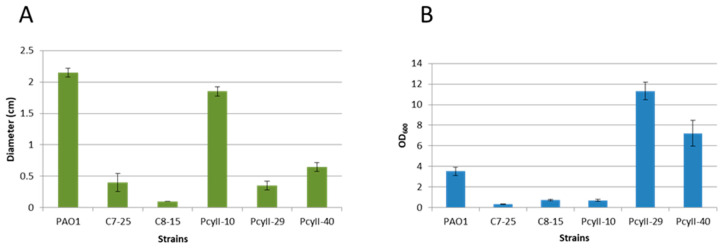
Phenotype of the strains. (**A**) Twitching motility is expressed as the diameter (cm) of the growth zone at the bottom of the agar plate. The standard deviation is the result of three independent assays. (**B**) Biofilm formation assay. The amount of bacteria bound to the wells is evaluated by measuring the OD_600_ of crystal violet resuspended in ethanol. The standard deviation is the result of three independent assays.

PcyII-10 (serotype O6), a highly susceptible strain, and four nontypable MPhR strains (PAC7-25, PAC8-15, PcyII-29 and PcyII-40) were selected for additional analyses. Multiple locus Variable number of tandem repeat (VNTR) analysis (MLVA) genotyping of these strains suggested that they belonged to different genetic clusters (data not shown) [3]. PcyII-29 and PcyII-40 formed rough colonies with sharp edges and displayed autolysis, whereas PAC7-25 formed small round colonies and PAC8-15 was mucoid (Figure S1). Upon overnight culture in Luria broth (LB) medium, floating cell aggregates were observed with PAC7-25 and PcyII-29. We then analyzed the motility of the bacteria, a complex behavior mediated by different extracellular structures. Type IV pili are essential for twitching and are involved in biofilm formation [51,52]. All phage-resistant strains showed reduced capacities to twitch (Figure 2A), suggesting that they were deficient in pili synthesis. Interestingly, the biofilm formation was high with PcyII-29 and PcyII-40 as compared to the other strains (Figure 2B). The capacity to swarm was altered in PAC8-15 and PcyII-40 as compared to PcyII-10 and was absent in PcyII-29 and PAC7-25 (Figure S2), suggesting a defect in the biosynthesis of pili, flagella or surfactant molecules, as previously reported [53].

### 2.2. Genome Sequence Characteristics

The genomes of three MPhR strains (PcyII-29, PcyII-40 and PAC7-25) and one sensitive strain (PcyII-10), all susceptible to antibiotics, were sequenced by a combination of PacBio and Illumina technologies, allowing the chromosome assembly into a single molecule. The size and some characteristics of the genomes sequenced in this work are indicated in Table 1. The three MPhR strains had genomes significantly larger than those of PcyII-10 and PAO1 (AE004091 size 6,264,404 bp). Alignment of the four genomes with MAUVE showed a similar architecture with locally collinear blocks (internally free from genome rearrangement [54]), except for an inversion of two regions shown with light blue and purple in Figure S3. The MLVA (Table S2) and Multilocus sequence typing (MLST) (Table S3) codes were deduced from the sequences, and clustering analyses were performed, confirming that the strains were genetically clearly distinct. Interestingly, in PAC7-25, four IS elements were present in VNTR loci used in the MLVA assay, which prevented amplification by PCR, as previously reported [3,55]. Coding sequence identification and annotation of the four genomes were performed, making use of data from published genomes. To question the mechanisms of resistance to phages, and since tens of genes may potentially be involved, we focused our analyses on regions known to possess genes responsible for the biosynthesis of primary receptors. We also investigated the accessory genomes encompassing prophages, genomic islands and other MGEs possibly associated with defense mechanisms. We compared the genome of the three resistant bacteria to that of PcyII-10 and to reference strains and investigated sequences presenting important differences.

### 2.3. The LPS and O-antigen Biosynthesis Genes

Biosynthesis of the three LPS domains, the lipid A, the core and the O polysaccharide A and B chains requires the expression of a large number of gene products [56]. The MPhR strains were nontypable and presented a deficiency in LPS production. In an attempt at finding the basis for this deficiency, we first analyzed genes responsible for the synthesis of the O-antigen, as described in [57] (Figure S4). A cluster of genes responsible for serotype O:6 O-antigen biosynthesis similar to that of strain LESB58 (NC_011770) [18] was found in both PcyII-10 and PAC7-25. The cluster was intact in PcyII-10, whereas in PAC7-25, one of the genes (PLES-19151 in LESB58) encoding a glycosyltransferase was interrupted by the insertion of a 1201 bp IS element (position 2,017,419). In strain PcyII-29, which possessed a cluster corresponding to serotype O:4, a 1239 bp IS was inserted into the same gene as in PAC7-25. In PcyII-40, the cluster corresponding to serotype O:11 appeared intact as compared to that of the reference serogroup O:11 strain PA103 [58]. We previously analyzed in detail the constitution of LPS in reference strain PAO1 and variants resisting different bacteriophages to uncover the effects of various mutations [39]. The structure of LPS in PcyII-40 as observed by PAGE analysis (Figure 1) was similar to that of a PAO1 mutant defective in Wzy, the glycosyltransferase responsible for linking groups of three sugars to form B-chains [39]. Such mutants lack long B chains and show the presence of high amounts of the core+ 1 subunit. However, the LPS defect in PcyII-40 could not be attributed to the Wzy protein, as it was identical to that of PA103 (Figure S5). Similarly, the *wzz1* and *wzz2* genes, both essential for very long B chain synthesis in strain PA103 [59], did not show modifications in PcyII-40 as compared to PA103 (PcyII-40 open reading frame (ORF) 4661 in genome LR739069 is equivalent to Wzz2 PAO10938).

LPS analysis previously revealed that PcyII-10 produced very long B chains and little long and medium chains, such as observed in strain PA14 and attributed in this strain to mutations in *migA* [60]. The mutation R92Q was found in PcyII-10 and in PA14 but not the second mutation, V170M, and there was no defect in production of the uncapped core in PcyII-10. PcyII-29 also showed the R92Q substitution.

Little variation was seen in the cluster of genes involved in core oligosaccharide gene synthesis, as described by Liebens et al. [61]. In PAC7-25 and PcyII-10, a gene encoding an Aspargine synthetase (PcyII10_5205 in genome LT673656) was found upstream of this cluster that was absent in the other two strains, but its role is not known.

### 2.4. Pili and Flagella

Over 40 genes distributed into several clusters at different locations in the genome are known to be involved in the T4P biosynthesis [62,63,64]. These genes were investigated in search for characteristics or mutations that could explain the apparent absence of functional T4P in the MPhR strains. In the *pilMNOPQ* cluster, a phase-variation mutation in PcyII-29 *pilN* predicted the production of a shorter protein. PilN and PilO dimerize and anchor the complex in the periplasmic membrane, where it becomes associated with other proteins to produce a transenvelope complex that interacts with PilA [65]. A group of three genes involved in the synthesis of fimbriae (two fimbrial proteins and a pilus assembly chaperone, ORFs 0499, 0500 and 0501 respectively in PcyII-10 genome LT673656) showed differences between PcyII-10 and the MPhR strains. The chaperone gene *fimC* was very different between PAC7-25 and PcyII-10, and one of the fimbrial protein (PcyII10_0500 in genome LT673656) was absent in PAC7-25 and PcyII-29, whereas the cluster of three genes was absent in PcyII-40 [66]. Minor differences were observed in the other genes, such as a 18-bp insertion in *chpA* in PcyII-40. This gene encodes a large chemosensory protein involved in motility [67].

### 2.5. The Accessory Genome: Regions of Genome Plasticity

Mathee et al. defined an RGP as any region of at least four contiguous ORFs that is missing in at least one other *P. aeruginosa* genome [11]. Using this definition, we identified genomic regions that were variably present in the analyzed strains. They corresponded mostly to MGEs (prophages, R-M systems and GIs), but a few clusters of genes were also differentially distributed. The three MPhR strains displayed numerous MGEs, the majority of which were absent in the phage-susceptible strains PAO1 and PcyII-10, which explained the difference in genome sizes. In addition to the presence of MGEs at known RGPs, we identified three insertion sites that were not previously described (RGP95, 96 and 97).

### 2.6. Prophages and Genomic Islands

We used IslandViewer to identify regions that most probably were MGEs [18]. We also searched specifically for prophages using PHASTER and combined the results of the two analyses in Table S4 (the major component of the island is indicated in the column “element”). Each region is called using the strain name and an MGE number. Among common features found in the four strains was the presence of a gene cluster at RGP 3+4 encoding a R2-type pyocin [68,69]. The locus was inside a group of genes involved in tryptophan biosynthesis, in between *trpE* and *trpG* [70]. Several other common sites (shown with the same color on Table S4) were shown to harbor different MGEs. One or two copies of Pf1-like phages were inserted at different positions in the PcyII-10, PAC7-25 and PcyII-29 genomes (Table S4). The genome of these prophages varied in size, as previously observed in other *P. aeruginosa* strains [71]. Similarly, phages with genomes close to that of the F10 siphovirus [72] were observed in PcyII-29 and PcyII-40, with noticeable differences. At the same position in the three F10-like phage genomes, before genes encoding a holin and an endolysin, PCYII-40_MGE3 possessed a HicAB-type toxin-antitoxin system (ORF PCYII40_0891 and PCYII40_0892 showing similarities with antitoxin YncN) [73], whereas PCYII-40_MGE4 had Asn-tRNA and thr-tRNA genes, and PCYII-29_MGE2, a group of genes of unknown function and an integrase.

PAC7-25_MGE1 contained a type I R-M system inserted together with a phi3-like (KT887559) prophage (PAC7-25_MGE2) adjacent to the Met tRNA gene. In PAC7-25_MGE4, a type II and a type III R-M system were found in a 24-kb island also containing several mobile elements. PAC7-25_MGE5 was a genomic island found in its wholeness in a single *P. aeruginosa* strain, LW (CP022478), and partially in a few other *Pseudomonas* species. PAC7-25_MGE6 corresponded to the 64-kb Pf725A prophage that we previously characterized as belonging to the F116-family of transposable phages (Genbank LT603684) [74]. PAC7-25_MGE7 contained a RelBE TA (toxin/antitoxin) addiction module. PAC7-25_MGE8 was a Pf1-like phage at position 5,564,441-5,574,343, a previously undescribed insertion site near the tRNA^Sec^ gene. We called this new site RGP95, corresponding to position 4802/4803 in PAO1.

Nine MGEs were found in PcyII-29 of which three were intact prophages: PCYII-29_MGE2, PCYII-29_MGE5 and PCYII-29_MGE8. PCYII-29_MGE3 was a 30-kb island including two phage integrases, a transposase, a reverse transcriptase and a type I R-M system (RGP96). No repeated sequences were identified at the junctions of these elements. PCYII-29_MGE4 corresponded to a genomic island observed in LESB58 (LESGI-2) and containing the naringenin-chalcone synthase gene followed by the cluster of genes for pyoluteorin biosynthesis, a polyketide antibiotic with a strong bactericide and fungicide activity [75]. It included the regulatory gene *pltZ*, previously found in *P. fluorescens*, and an ABC transporter, as in *Pseudomonas* sp. M18, which plays a role in self-resistance against the toxic effect of pyoluteorin [76]. It was previously found in *P. aeruginosa* strain LESB58 at the same position. PCYII-29_MGE6 was a 78-kb island encompassing a prophage tail biosynthesis gene cluster, and PCYII-29_MGE7 was a 56-kb genomic island encompassing a type I R-M system, helicases, kinases and genes of unknown functions, with several phage integrases and transposases. PCYII-29_MGE8 combined the PAGI-6 genomic island and a copy of a phiCTX-like phage. Finally PCYII-29_MGE9 corresponded to a 103-kb pKLC102-like ICE originally described in clone C strains [77,78]. It is inserted next to the lys tRNA structural gene, as previously observed in other *P. aeruginosa* isolates [78,79]. Among its 110 ORFs, this ICE possessed a cluster of T4P genes and the *ndvB* gene conferring biofilm-specific antibiotic resistance.

In PcyII-40, 15 MGEs could be observed, including seven intact prophages and an R-plasmid. PCYII-40_MGE13 corresponded to the insertion of a mu-like phage (phage PfII40a) at RGP 97 that we previously described [80]. Among the other prophages, two were F10-like, one was phiCTX-like and one was pf1-like. Two phages were completely new, PCYII-40_MGE5 and PCYII-40_MGE8. The PCYII-40_MGE8 genome showed some similarities with phage AF and Ab31 [25] and had 83% similarity over some part of its genome with phage ctdb7, a putative podovirus with a 42,196-bp-long genome identified in an animal virosphere by a metagenomics approach (MH593831). Two type I R-M systems were present at PCYII-40_MGE1 and PCYII-40_MGE12. PCYII-40_MGE6 held a cluster of genes responsible for conjugative transfer and cation efflux system genes. PCYII-40_MGE7 possessed genes responsible for the transport and efflux of different molecules and an S2 pyocin (ORF 3128 in genome LR739069). PCYII-40_MGE9 possessed another cluster of genes responsible for conjugative transfer, as well as genes encoding efflux transporters. In addition to the regions found by IslandViewer, we detected a cluster of four genes (located at positions 1,273,527 to 1,278,865) encoding a pyocin-S2-associated to the DNA recombinase e14, an integrase and an exeA-like protein. Another pyocin-S2 (ORF 4429) and its immunity protein (ORF 4428) were found at positions 4,731689 to 4,734023 [81]. By Blast, the pyocin matched HNH nucleases of the colicin family.

### 2.7. CRISPR-Cas Systems

In PcyII-10, three Type I-F CRISPR arrays with the same 28-bp repeat were identified using CRISPRCasFinder [82]. CRISPR1 and CRISPR2 were clustered at RGP12, at positions 2,784,771–2,785,758 (20 repeats) and 2,794,282–2,795,509 (23 repeats), respectively, separated by a group of six *cas* genes, as previously observed [34], whereas CRISPR3 was at position 1,488,702–1,490,109 (24 repeats). No CRISPR-Cas system was observed in PAC7-25 and PcyII-40. PcyII-29 possessed a single CRISPR array, with sixteen repeats at position 1,522,234–1,523,041 (corresponding to CRISPR3 of PcyII-10). The 28-bp consensus repeat was that of the *P. aeruginosa* type I-F system. In this strain, CRISPR1, CRISPR2 and the *cas* genes were absent, and there was no other deleted sequence at this position.

### 2.8. Transposases and IS

We used IS saga and MicroScope to identify regions encoding transposases. A large number of transposases and IS elements often associated with RGPs were found in the three MPhR strains and were absent in PcyII-10 (Table S5). Members of the IS3 and IS9 families were found in the four genomes, whereas each strain possessed a particular family of IS, sometimes in high copy numbers.

In PAC7-25, 14 copies of a 1017-bp IS110 element (related to ISPa11) were present, but none were detected in the other strains. IS110 copies were inserted into four VNTRs (ms142, ms211, ms212 and ms216), as observed by an in silico MLVA analysis. PAC7-25 also specifically possessed an IS5-like element and an IS200-like element. Five copies of an IS3-like element (detected in two consecutive fragments) were observed, three of them being associated with a genomic island. IS3-like elements were also found in the other strains, some inserted at the same site.

In PcyII-29, a large number of IS3-like sequences with homologies to IS222, a 1234-bp *P. aeruginosa* IS, were found [83,84]. Several of them were present together with other IS and with an R-M system at RGP6 and RGP96. Tn3 elements were detected at RGP23 in PCYII-29_MGE6.

PcyII-40 also held multiple IS3-like elements, as well as ISL3 and IS481. These elements were associated with R-M systems, with a plasmid and with pyocin-S2. Tn3 were present with plasmid Rms148 (PCYII-40_MGE7).

### 2.9. Activation of Prophages and Phage Tail Pyocins

**Figure 3:**
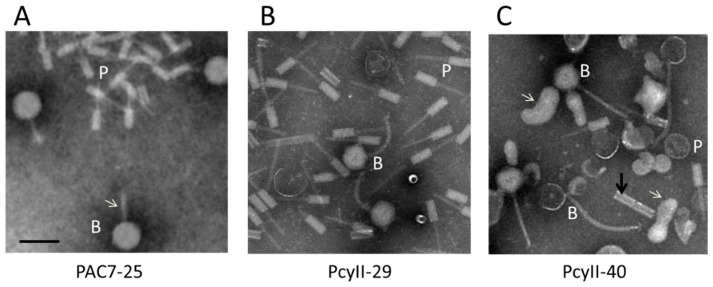
Electron microscopy analysis of particles induced by mitomycin C. (**A**) PAC7-25 where the white arrow points to the phage tail, (**B**) PcyII-29 and (**C**) PcyII-40 where the white arrows point to vesicles, and the dark arrow points to a group of two attached ~50-nm tubes. Pyocins are indicated with a P and bacteriophages with a B. The black bar represents 100 nm.

In order to identify viable prophages in the three MPhR strains, bacterial cultures were treated with mitomycin C (MitC) during exponential growth, resulting in rapid and massive lysis. The culture supernatant was enriched in phage particles for electron microscopy (EM) examination and for DNA purification and sequencing. Upon EM examination of the PAC7-25 supernatant, large amounts of phages with large heads (~77–78 nm) and what appeared to be thin tails (shown with an arrow) and R-type pyocins were observed (Figure 3A). The PcyII-29 supernatant contained siphoviruses with a ~68-nm head and a ~150-nm flexible tail, as well as R-type pyocins (Figure 3B). For PcyII-40, at least two different tailed phages were observed by EM with large amounts of vesicles, R type pyocins and ~50-nm tubes attached in groups of two to four (Figure 3C). The presence of vesicles could be related to explosive cell lysis mediated by the endolysin of R pyocins, as previously described [85]. Such vesicles are vectors for multiple molecules and DNA.

**Figure 4:**
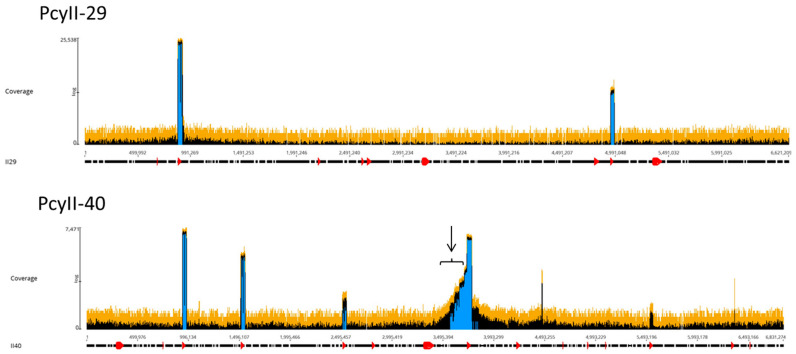
Mapping of sequencing reads from MitC-induced virions onto bacterial genomes. About one million reads were used in Geneious PcyII-29 and PcyII-40. The arrow points to reads mapping on chromosomal DNA.

**Figure 5:**
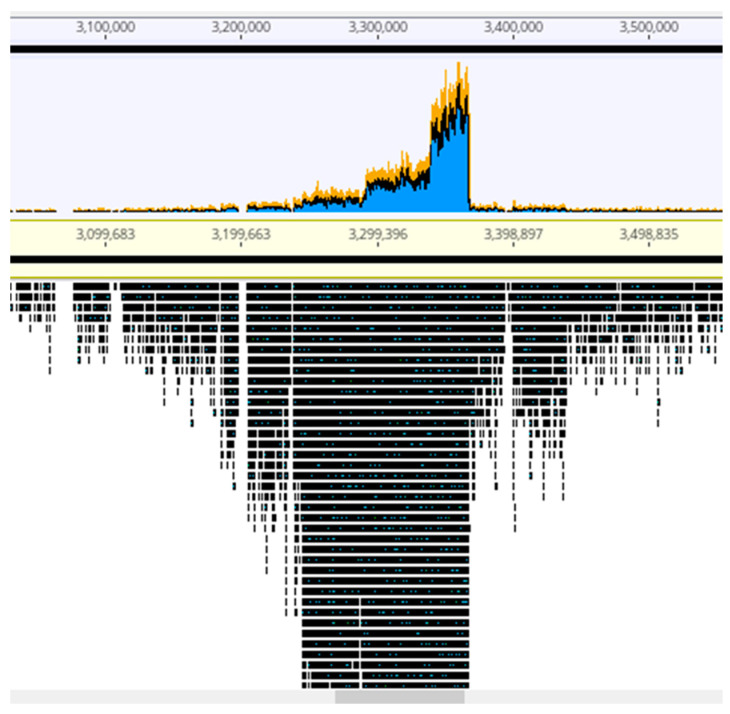
Mapping of the MitC-induced phages’ reads from PcyII-40 onto the PAO1_Or_ chromosome. A portion of the chromosome corresponding to the insertion site of phage PCYII-40_MGE8 is shown. The larger peak of reads is at the left side of the *attB* site and covers about 30 kb. Three additional peaks covering 46kb are observed.

The viral particles were digested with DNase 1 to eliminate contaminating cellular DNA followed by lysis, DNA purification and sequencing. For each sample, the Illumina sequencing reads were mapped onto the corresponding bacterial sequenced genomes, revealing the existence of peaks of activated prophages. For PAC7-25, a single phage was activated corresponding to Pf725A. Figure 4 shows the mapping of the reads from the PcyII-29 and PcyII-40 MitC-activated virions along the genome of their host (PcyII-29 and PcyII-40, respectively). For PcyII-29, peaks of reads were observed for phages inserted at PCYII-29_MGE2 (F10-like) and PCYII-29_MGE8 (phiCTX-like), corresponding to 97% and 0.8% of total reads, respectively (Table S6). Some bacterial DNA was present and aligned over the whole genome sequence. For PcyII-40, five peaks corresponded to prophages PCYII-40_MGE3, PCYII-40_MGE4, PCYII-40_MGE5, PCYII-40_MGE8 and PCYII-40_MGE13 with, respectively, 52%, 6.6%, 0.1%, 35.5% and 0.026% of reads (Table S7). We could assemble the reads to a single contig for these different activated phages, and they allowed defining the precise insertion site in the bacterial chromosome. Interestingly, 2% of bacterial reads present in the MitC-induced phage particles appeared to map mostly on one side of the phage PCYII-40_MGE8 insertion site (ATCACCATGCAGTT), extending over 170,000 bp. We then mapped the reads onto the PAO1_Or_ genome (Genbank LN871187), which lacks the PCYII-40_MGE8 prophage (Figure 5). It allowed distinguishing a high peak covering 30kb and starting at the precise position of *attB* (3,367,309) and, on its left side, three additional peaks of decreasing intensity, each extending over 46kb, the size of the phage genome. A small proportion of reads was present on the other side of the prophage insertion site. The existence of these bacterial sequences in MitC-activated virions is compatible with the packaging of DNA of the phage size, starting at a viral *pac* site localized about 16kb from the insertion site during the process of full-head packaging. At this position, we found the motif GCTAAA at the end of the gene encoding the terminase, similarly to the *pac* site described for SaPi in *Staphylococcus aureus* [86].

In order to try and recover the activated prophages, the supernatant of the MitC-induced cells was tested by spot assay on a large collection of strains. Pf725A, the F116-like phage spontaneously released from PAC7-25, could grow on PcyII-10 and PAO1_Or_ [74]. For PcyII-29, a single phage called PfII29a producing very small clear plaques could be obtained on strain PcyII-36 and corresponded to a F10-like phage (PCYII-29_MGE2), as confirmed by PCR amplification and sequencing. Attempts to isolate the phiCTX-like phage (PCYII_29_MGE8) failed. Although four phages appeared to be highly activated in MitC-induced PCYII-40, as shown by the peaks of reads in Figure 4, only three different phages were obtained from the supernatant. We identified a mu-like phage (PfII40a) growing on reference strain PA14 and on PcyII-10 (small, clear plaques with a halo) and two F10-like phages, PfII40b (PCYII-40_MGE3) and PfII40c (PCYII-40_MGE4), growing on PAO1 and PcyII-33 respectively. PfII-40c also grew on strain PAC9-6, making turbid plaques after 24 h that enlarged and became clear 48 h after infection. PfII40a (PCYII-40_MGE13) was previously isolated from spontaneous induction (Genbank LT608331). The integration of the PfII40a genome into the bacterial chromosome during the productive replication cycle is described in more detailed in [80]. The phage PCYII-40_MGE8 appeared to be highly induced based on the number of reads, but we failed to recover this phage following the infection of 20 different *P. aeruginosa* strains.

## 3. Discussion

The present results suggest that what makes *P. aeruginosa* strains resistant to multiple phages is the existence of a large accessory genome encoding genes for several resistance mechanisms and a variety of prophages. The PcyII-40 mu-like phage present at PCYII-40_MGE3 might be capable of superinfection exclusion but we don’t know whether such a mechanism is provided by any other prophage. These strains also possess genes that allow them to attack and destroy other *P. aeruginosa* strains. The cluster of genes in PCYII-29_MGE4 allows the synthesis of pyoluteorin, an antifungal compound [75]. *P. aeruginosa* strains bearing multiple MGEs were previously described, but this characteristic was not linked to the capacity to resist phages [9,87].

IS were numerous in MPhR strains, some being responsible for inactivating genes, leading to a loss of function and, in particular, in LPS and T4P biosynthesis. In some *P. aeruginosa* strains, movements of IS were shown to be particularly frequent, thus enhancing the capacity of the cells to mutate their genes in a reversible fashion [88]. This might be very important for survival in the environment, where the integrity of the membrane and presence of appendages are essential. Among the genomic islands, those conferring innate immunity were abundant, with two or three different R-M systems in each strain, offering a variety of targets [89,90]. It is interesting to observe that R-M systems and multiple prophages can be found in the same cell, reflecting phage-host population dynamics and confirming, as previously proposed, that R-M can promote prophage acquisition at the population level [91]. Indeed, the three MPhR strains were polylysogenic, in particular PcyII-29 and PcyII-40 which possessed a large variety of prophages, some showing no similarity with known viruses at the genome level. Sequencing of the MitC-activated prophages showed that different levels of expressions between phages were achieved, some being produced in very large amounts. Among those that could be cultivated, F10-like phages have the largest host range, and they presented some interesting characteristics. A comparison of their genomes showed blocks of highly conserved sequences alternating with diverged regions holding genes that participate in virulence.

At least four phages were activated in PcyII-40, of which PCYII-40_MGE8 was shown to perform lateral transduction by packaging the chromosomal DNA present at the site of the prophage insertion. This behavior was first described with phage Pf725A and later observed with *Staphylococcus aureus* phages [74,92]. The proportion of packaged bacterial DNA to phage DNA in Pf725A was smaller than with PCYII-40_MGE8 [74]. This category of phages is particularly interesting, as they are responsible for transducing bacterial DNA with a high frequency [93]. The host range of phage PCYII-40_MGE8 is very restricted, and we could not find a host to amplify it, therefore limiting further investigations.

The presence of CRISPR-Cas systems was negatively correlated with the presence of MGE, as only the phage-susceptible strain PcyII-10 possessed an intact CRISPR-Cas system. This is in agreement with previous observations suggesting that this defense system is not a major mechanism of resistance to lytic phages [34], although the CRISPR arrays occasionally carry spacers targeting lytic phages [35]. In PcyII-10, the CRISPR-Cas system may have played a role in limiting the accumulation of IS, although no spacers in the three CRISPR arrays matched these or other PcyII-10 genomic sequences. It was proposed that the presence of self-targeting spacers resulted in a loss of MGE-bearing protospacers [94].

Immunity provided by prophages may not be the most important mechanism for multiphage resistance, as polylysogeny is frequent in *P. aeruginosa*, and most temperate phages show a narrow host range and rarely provide heteroimmunity [95,96]. However, some prophages encode a variety of functions that participate in the defense against viral attacks, as shown in *P. aeruginosa* for mu-like bacteriophages [23] and for *Mycobacterium smegmatis* [97]. By comparison with observations made with mu-like phage D3112, PfII40a, a related phage [80], may confer heteroimmunity through different mechanisms, such as the specific interaction between the c repressor and the cognate operator [98]. Pf1-like bacteriophages were found in all the strains. In strain LES (Liverpool Epidemic Strain), these phages are highly active and may have a role in the competitiveness of LES [18,99,100,101]. They contribute to bacterial short-term evolution and virulence [27].

The three strains lack LPS O-antigen B bands and functional type IV pili. T4P are involved in multiple cellular functions [102] and constitute an important virulence factor of *P. aeruginosa* [103]. As a consequence of mutations in T4P biosynthesis genes, the motility of the MPhR strains was strongly affected, but two of the investigated strains, PcyII-29 and PcyII-40, still formed biofilms with a high efficiency, a factor that may help them escape antibiotics.

It is important to stress that the three strains selected on the basis of their resistance to multiple phages turned out to be those that possess the highest number of prophages as compared to PAO1 and PcyII-10. They spontaneously release phages when grown to the plateau or on richly infected plates. Their capacity to release R-type pyocins and phages may give them a competitive growth advantage over negative *P. aeruginosa* strains [69,104] and may contribute to the regulation of *P. aeruginosa* density in vivo, as suggested [105].

## 4. Materials and Methods

### 4.1. Ethics Statement

The present project is in compliance with the Helsinki Declaration (Ethical Principles for Medical Research Involving Human Subjects). Bacterial strains were collected as part of the patients’ usual care, without any additional sampling, as previously reported [55]. The ethic committee “Comité Consultatif pour la Protection des Personnes dans la Recherche Biomédicale (CCPPRB) Ile-de-France”, who was consulted, specifically approved this study and declared that patient informed consent was not needed.

### 4.2. Strains and Media

Strain PAC7-25 (nontypable, genetically O6) and PAC8-15 were isolated from cystic fibrosis patients and were previously investigated as part of the projects on the mechanisms of antibiotic resistance [55] and susceptibility to bacteriophages [34]. PcyII-10 (O6), PcyII-29 (nontypable, genetically O4), PcyII-40 (nontypable, genetically O11) and 24 additional clinical strains were isolated from burn patients in Hopital de Santé des Armées Percy, Clamart, France. The bacteriophages used in this study belong to ten different genera (Table S1) and were previously described [48,106,107]. Luria broth (LB) medium supplemented with 2-mM CaCl_2_ was used for bacterial growth. Phosphate-buffered saline (PBS) was used for phage titration and to preserve purified phage at 4 °C. Lipopolysaccharides (LPS) were purified using the method of Hitchcock and Brown [108]. The LPS were resolved by electrophoresis on a 15% SDS-polyacrylamide gel, and the band pattern was visualized using the silver staining method [109].

### 4.3. Prophage Induction and Purification

To induce prophages, 20-mL LB were inoculated at 1/100 with an overnight bacterial culture and shaken at 37 °C until the culture reached an OD_600_ of 0.6. Mitomycin C was added to a final concentration of 3µg ml^−1^, and the incubation was continued until lysis occurred (3 h at 37 °C). Culture supernatant was tested for the presence of bacteriophages by spotting five µl of sequential dilutions on a lawn of sensitive bacteria in soft agar. Single plaques were recovered and purified by three successive platings. The newly isolated phages were amplified for eight hours on solid medium by mixing 10^9^ colony forming units (c.f.u.) of bacteria with 10^6^ plaque-forming units (p.f.u.) per plate, as previously described [25]. Bacteria and debris were pelleted by centrifugation, the supernatant was recovered and phages were precipitated with 6% polyethylene glycol (PEG) 8000 overnight at 4 °C. After centrifugation at 15,000× *g* for 20 min, the pellet was suspended in one ml of PBS and treated for two hours with DNase1 (50 µg mL^−1^) at 37 °C. Three chloroform extractions were performed prior to filtration through a 0.45-µM filter and centrifugation for two hours at 260,000× *g*. The pellet was suspended in 50 µL of PBS. For electron microscopy (EM) visualization, five µl of phage suspension was stained with 2% potassium phosphotungstate (pH 7.0), as previously described [34].

### 4.4. Phenotypic Assays

#### 4.4.1. Swarming Motility

One microliter of overnight bacterial culture was spotted on agar plates freshly prepared (one hour before depositing the bacteria) using “swarming media” containing 0.5-mM MgSO_4,_ 0.75-mM CaCl_2_ and 10.5 g of M9 Broth (Fluka Analytical, Switzerland); 2-g dextrose; 5-g casamino acids (MP, USA); 5-g agar per liter. The growth was measured after 48 h.

#### 4.4.2. Twitching Motility

One µl of overnight bacterial culture was inoculated between the agar and the plastic surface of 1.5% LB agar plates. The diameter of the motility zone around the inoculation site was measured after 24 h incubation at 37 °C.

#### 4.4.3. Biofilm Formation

Overnight cultures were diluted at an OD_600_ of 0.5 and used to fill a 96-well plate (200 µL per well). The plate was incubated at 37 °C for 70 h. The OD_600_ of the bacterial growth was recorded prior to a crystal violet assay (blank = LB media). The plate was washed once with PBS, and then, 300 µL of crystal violet 0.1% was added and kept for 10 min at room temperature. The unabsorbed crystal violet was washed twice with PBS, and 200 µL of ethanol 100% were added to each well before OD_600_ was measured (blank = ethanol 100%) using the CLARIOstar microplate reader. The assay was performed making seven replicates per each strain. The first and last lines were not considered for the determination of the biofilm formation. The final score of each strain for the biofilm production was the result of the division between the final crystal violet measurement and the bacterial OD_600_ measured before the crystal violet treatment.

### 4.5. DNA Extraction and Analysis

Samples were lysed in lysis buffer (Tris 10 mM, pH 7.8, EDTA 10 mM, NaCl 10 mM and SDS 0.5%wt/vol) and treated with proteinase K at 50 µg mL^−1^ for 2 h at 50 °C, followed by one phenol and one chloroform extraction and ethanol precipitation. Genotyping of the bacterial strains was performed by multiple locus VNTR analysis (MLVA), as described [3]. Briefly, the VNTR loci were PCR-amplified, and the products were analyzed on agarose gel by electrophoresis. The presence of an IS element of the size 1kb or more produced large amplicons or prevented correct amplification.

### 4.6. Whole Genome Sequencing and Annotation

Draft whole genome sequencing was performed by the high-throughput sequencing core facility of I2BC (Centre de Recherche de Gif—http://www.i2bc.paris-saclay.fr/). Libraries were made from sheared fragments of DNA (average size 900 bp), and 250-bp or 300-bp paired-end reads were produced on the MiSeq platform (Illumina, CA, USA). Pacific Bioscience SMRT (PacBio, CA, USA) technology and the de novo hybrid assembly from PacBio and Illumina sequence data were applied to four genomes and were performed by BaseClear (Leiden, The Netherlands). The Illumina FASTQ sequences were trimmed off low-quality bases and de novo assembled using CLC Genomics Workbench version 8.0 (GIAGEN, Aarhus, Denmark). The optimal k-mer size was automatically determined using KmerGenie [110]. The contigs were linked and placed into super-scaffolds based on the alignment of the PacBio CLR reads using the SSPACE-LongRead scaffolder version1.0 [111]. Alignment was performed with BLASR [112]. The gapped regions within the super-scaffolds were partially closed in an automated manner using GapFiller version 1.0 [113]. Final gap closure was achieved when necessary with the help of Geneious R9 (Biomatters, Auckland, New Zealand). Annotation was done using MaGe at the Microbial Genome Annotation and Analysis platform (MicroScope) [114], RAST http://rast.nmpdr.org/ [115] and BASys https://www.basys.ca/ [116]. PHASTER http://phaster.ca [117], a better version of PHAge Search Tool (PHAST) [118], was used to identify the presence of phage sequences. The core and accessory genomes were identified using the Spine and AGEnt bioinformatics tools [119]. GIs were identified by IslandViewer [120]. Insertion sequences (IS) and transposons were detected using IS saga http://issaga.biotoul.fr/. In silico MLVA was performed using scripts available at https://github.com/i2bc/MLVA_finder. The MLST type was deduced from the sequence and established using the PUBMLST website http://pubmlst.org/ and the MLST plugin for BioNumerics 7.6.3 (Applied-Maths, Sint-Martens-Latem, Belgium). All clustering analyses were performed within BioNumerics 7.6.3. MLVA allele numbers expressed as a numerical code were clustered using the categorical coefficient and the Unweighted Pair Group Method with Arithmetic mean (UPGMA) algorithm, producing a dendrogram, as previously described [3]. Strains inside a MLVA cluster shared 12 alleles out of 14. Strains inside a MLST cluster shared 6 alleles out of 7 [1]. The CRISPR-Cas loci were identified using CRISPRCasFinder at http://crisprcas.i2bc.paris-saclay.fr/ [82]. Whole genome alignments were performed with MAUVE in Geneious R9. MAUVE identifies conserved segments that appeared to be internally free from genome rearrangements [54]. Such regions are referred to as locally collinear blocks (LCBs).

Phage genome sequencing was done using the I2BC MiSeq Illumina sequencing platform. Phage genome assemblies, sequencing reads mapping to genome references, prophage insertion site identifications and other sequence analyses were done using the tools in Geneious R9. The assembly tool was Geneious assembler.

### 4.7. Nucleotide Sequence Accession Number

The complete genome sequences of the four bacterial genomes PcyII-10 (accession LT673656), PcyII-29 (LR739068), PcyII-40 (LR739069) and PAC7-25 (LR739071) are available from the European Nucleotide Archive (ENA) browser at http://www.ebi.ac.uk/ena/data/view/<ACCESSION NUMBERS>. The raw sequence data have been deposited in projects PRJEB18612 and PRJEB35547. Two prophage sequences were previously described: pfII40a (accession LT608331) [80] and phiC725A (accession LT603684) [74], induced from PcyII-40 and PAC7-25, respectively.

## 5. Conclusions

Phage therapy has been used with success to treat infections by multidrug resistant bacteria and may be more widely applied in the future [121]. Long-term infections in CF and burn patients often involve strains that are susceptible to antibiotics in vitro but form biofilms and cannot be eradicated, therefore requiring alternative therapeutic approaches [122]. Unfortunately, some of these strains resist most of the available phages, and this calls for the optimization of known bacteriophages, the continuous search for new viruses [123] and a better understanding of the basis for resistance. A resistance to phages is generally not considered to be a problem, because it is expected to strongly affect the bacterial fitness, leading to a rapid outgrowth by susceptible bacteria. We showed here that, despite an accumulation of factors that should decrease their chances of survival in the environment, resistant strains also presented clear advantages that allowed them to establish long-term infections. A combination of treatment by phages and antibiotics will be necessary to eradicate such strains.

## Figures and Tables

**Table 1:** Characteristics of the sequenced genomes.

Strain	Genome ID	Size (bp)	%GC	CDS *	tRNA *	Serotype	ST *	Closely Related Genome **
**PcyII-10**	LT673656	6,288,645	66.50%	5787	64	O6	1233	**LESB58** **(NC_011770)**
**PAC7-25**	LR739071	6,479,881	66.30%	5955	65	O6	1710	**YL84 (CP007147)**
**PcyII-29**	LR739068	6,621,209	66.30%	6156	65	O4	175	**F22031 (CP007399)**
**PcyII-40**	LR739069	6,831,274	66.10%	6395	66	O11	309	**UCBPP-PA14 (CP000438)**

* CDS, coding sequence; tRNA, transfer ribonucleic acid; ST, sequence type as defined in the *P. aeruginosa* PubMLST database https://pubmlst.org/paeruginosa/ ** 99% identity over 90% to 98%.

## Supplementary Materials

The following are available online at https://www.mdpi.com/2079-6382/9/6/339/s1: Figure S1. Colony morphology of four MPhR strains. Figure S2. Swarming capacity of four MPhR strains and of PAO1 and PcyII-10. Figure S3. Whole genome alignment using MAUVE. Figure S4. Structure of the O-antigen gene clusters and comparison with type reference genomes. Figure S5. Result of submission to Blastp of the PcyII-40 Wzy protein sequence. Table S1. Phages used in the present study. Table S2. MLVA codes deduced using tools at: http://mlva.u-psud.fr. Table S3. MLST codes deduced using tools and data from: http://pubmlst.org. Table S4. Prophages and Conjugative elements as found using PHASTER and IslandViewer. Table S5: Insertion elements as encoding transposases using IS Saga. Table S6. Mitomycin C induction of PcyII-29. Table S7. Mitomycin C induction of PcyII-40.
